# Rapidly Polymerizing Click Hydrogel Provides Localized Delivery of rhBMP2 to Promote Bone Formation

**DOI:** 10.1002/prp2.70119

**Published:** 2025-06-08

**Authors:** D. Joshua Cohen, Thomas W. Jacobs, D. Scott Wilson, Michael C. Mancini, Christine Van Duyn, Zvi Schwartz, Barbara D. Boyan

**Affiliations:** ^1^ Department of Biomedical Engineering Virginia Commonwealth University Richmond Virginia USA; ^2^ Johns Hopkins Medical Institute Baltimore Maryland USA; ^3^ Pascal Medical Corporation Richmond Virginia USA; ^4^ Department of Periodontology University of Texas Health Science Center at San Antonio San Antonio Texas USA; ^5^ Wallace H. Coulter Department of Biomedical Engineering Georgia Institute of Technology Atlanta Georgia USA

**Keywords:** BMP, bone, bone morphogenetic protein‐2, click chemistry, hydrogel, in vitro, in vivo, osteogenesis, osteoinduction

## Abstract

Delivery of bioactive agents to achieve tissue regeneration at targeted sites with minimal side effects requires the use of biodegradable carriers and sustained release of the therapeutic at appropriate concentrations. We developed a copper‐free polyethylene glycol‐based click hydrogel to deliver bone morphogenetic protein‐2 (BMP2), a potent regulator for bone regeneration that is currently delivered to orthotopic sites using an absorbable collagen sponge, leading to a burst release of BMP2, potentially causing ectopic bone formation. In contrast, the hydrogel is delivered as a liquid, conforming to the contours of treatment sites, polymerizing rapidly at body temperature without generating heat, exhibiting minimal swelling after gelation, and releasing its payload as it degrades. We assessed the safety and effectiveness of BMP2 delivery in vitro and in mouse cranial defects in vivo, comparing it to BMP2 delivered via a collagen sponge. No toxicity was observed in vitro or systemically, nor was there allergic sensitization caused by the hydrogel in rabbits. Released BMP2 increased the production of osteogenic markers in vitro. Hydrogel + BMP2 caused equivalent defect closure and total bone growth compared to collagen + BMP2; however, there was more vascularization within the defect but less bone growth outside of the defect on the calvaria for hydrogel + BMP2 compared to collagen + BMP2. In conclusion, the click hydrogels used in this study are safe and effective for administering BMP2 with fewer undesired off‐target effects and high potential to be used with BMP2 for bone regeneration, supporting the use of click chemistry hydrogels to deliver bioactive agents to treatment sites safely and effectively.

## 1 Introduction

A hurdle in regenerative medicine has been localized delivery of therapeutic agents to a target site, minimizing systemic exposure and off‐target effects. Conventional methods of drug delivery often suffer from poor target specificity, leading to systemic side effects and low therapeutic effectiveness. To overcome these limitations, novel drug delivery systems have emerged that can selectively deliver therapeutic agents to specific tissues and cell populations, allowing controlled, spatiotemporal release of drugs at the site of action [1, 2].

One approach for achieving localized delivery of therapeutic molecules is to link them to biocompatible scaffolds that are implanted in the intended site [3, 4]. Their success relies on the release of the bioactive component by hydrolysis or resident tissue enzymes such as matrix metalloproteinases, or actions of the cells that migrate onto the scaffold [5, 6]. These scaffolds are often designed to permit cell attachment, and depending on the functional group(s) present on the scaffold, to differentiate along specific lineages [7, 8]. To further enhance the value of the scaffold for tissue regeneration, it may be constructed as a composite with other biopolymers, synthetic materials, or minerals, to achieve specific mechanical properties needed for optimizing cell response [7, 9, 10, 11, 12].

While composite biomaterials have emerged as a powerful approach to enhance the properties of drug delivery scaffolds for regenerative medicine, their complexity has made them less attractive as mechanisms for in situ delivery of pharmaceuticals or biologics, particularly where the goal is not dependent on providing a physical scaffold for new tissue formation. An alternative approach is to use hydrogels, which are three‐dimensional networks of hydrophilic polymers, as a drug delivery system. Click chemistry hydrogels offer numerous advantages as promising biomaterials for bone regeneration. Their efficient crosslinking via click reactions enables rapid gelation within seconds to minutes, making them ideal for delivery through injection and in minimally invasive surgical procedures. This rapid gelation minimizes undesirable diffusion of therapeutic agents from the injection site and ensures localized delivery.

The versatility of click hydrogels lies in their diverse click chemistries, each offering distinct advantages [7, 8, 9, 13, 14, 15, 16, 17, 18]. The widely used copper(I)‐catalyzed azide‐alkyne cycloaddition (CuAAC) offers rapid gelation and exhibits a high tolerance toward biological conditions. The most widely used click reaction for hydrogels, CuAAC readily links azide and alkyne functional groups under mild conditions. Thiol‐maleimide conjugation is a rapid and specific click reaction that can be used for hydrogel synthesis under physiological conditions. This reaction is particularly beneficial for encapsulating sensitive biological agents like cells or proteins. Thiol‐ene click chemistry is another efficient click reaction that offers good biocompatibility and can be combined with other functional groups. Thiol‐ene click reactions enable facile control over gelation kinetics and network properties. Beyond these prominent examples, various other click reactions, including cycloadditions, Diels‐Alder reactions, and photoclick reactions, are being investigated for hydrogel formation in regenerative medicine applications. Each click chemistry offers unique advantages and challenges, prompting researchers to optimize the choice based on specific needs. The modularity of azides and alkynes allows easy conjugation of targeting ligands, drugs, or cell adhesion peptides onto the polymer backbones [16, 19]. However, potential leaching of copper residues from the hydrogel can exhibit cytotoxicity and requires removal of copper ions for in vivo application.

To address this, copper‐free click reactions, such as strain‐promoted azide‐alkyne cycloaddition (SPAAC), are being explored as alternative biocompatible approaches. This metal‐free click reaction eliminates the need for copper removal/the potential cytotoxicity associated with copper catalysts, making it more suitable for biomedical applications. Biocompatible thiol‐ene/yne click chemistries offer rapid gelation and facile control over gelation kinetics and network properties. Another popular click reaction for hydrogels is thiol‐maleimide conjugation, which offers rapid and specific coupling under physiological conditions without any metal catalysts. This reaction is particularly beneficial for encapsulating sensitive biological agents like cells or proteins.

We hypothesized that copper‐free click chemistry could be used to develop a hydrogel for controlled delivery of biologics to bone sites following surgery [20, 21]. To obtain a rapidly polymerizing hydrogel, we synthesized PEG‐N3 through RAFT polymerization using azide functionalized and non‐functionalized PEG methacrylate monomers. This resulted in a polymer with tightly controlled azide functionality. The bifunctionalized PEG‐DBCO crosslinker was obtained by reacting bis‐amino‐PEG with benzyl‐2‐nitro‐carbonate functionalized DBCO. Unlike the conventional click method that relies on a Cu(I) catalyst, which is toxic in biological systems, the resulting hydrogel uses strain‐promoted alkyne azide cycloaddition (SPAAC). SPAAC operates swiftly at room temperature and eliminates the need for a toxic Cu(I) catalyst.

Our initial studies were designed to assess gelation, biocompatibility, and bioactivity of incorporated therapeutic agents in orthotopic sites. We first tested the hydrogel in a resynostosis model in weanling mice. The main goal of developing a resynostosis therapy was to delay the growth of new bone after craniosynostosis surgery in infants, allowing the bone to regrow naturally as the child grows older. The hydrogel was designed to be injectable and to crosslink in situ to completion in less than 2 min. To meet these requirements, multivalent poly(ethylene glycol) precursors were synthesized that formed an insoluble network upon mixing via SPAAC click chemistry reaction between dibenzylcyclooctynes (DBCO) and azides. This hydrogel system contains ester linkages to ensure that the system is ultimately degraded and excreted.

Bone formation was recorded via micro‐CT and histomorphometry, and histology of the underlying dura revealed no toxicity. We observed that hydrogel alone resulted in delayed resynostosis, and this could be extended by incorporation of Gremlin1, which is an inhibitor of bone morphogenetic protein 2 (BMP2), and the effect was dose‐dependent [20]. Based on the finding that the craniotomy defects healed by a process that depended on vascularization, we also tested the effectiveness of incorporating anti‐angiogenic compounds, including the anti‐VEGFA‐antibody and hypoxia‐inducible factor 1‐α inhibitor topotecan [21]. The payloads were added to the resulting PEG‐N3 copolymer component in liquid form and then injected into the craniotomy defect along with the DBCO crosslinker. The gelation process occurred within 90 s in vivo at body temperature, and resulted in a conformal gel that filled the bony contours created during suture removal. More recently, we examined the delivery of the neuropeptide semaphorin 3A to a femoral bone defect in adult rats in order to enhance bone formation around a titanium implant [22, 23], and using a bone implant onlay model in the cranium [24]. In addition to confirming the effectiveness of the hydrogel as a carrier system for a bioactive agent, the results of this study demonstrated that there was limited swelling of the hydrogel during and after gelation.

These studies showed that the hydrogel could be used to effectively deliver therapeutic agents to a target site, but they did not address the question of whether the hydrogel would retain bioactive molecules at the site and reduce off‐target effects. To get a more complete understanding, we took advantage of the extensive use of BMP2 as an osteoinductive agent in preclinical models [25, 26, 27] and clinically as the FDA‐approved product “Infuse” (Medtronic Spine, Memphis, TN). BMP2 is an osteoinductive growth factor that is used clinically to promote bone formation, regeneration, and fusion. Recombinant human BMP2 (rhBMP2) is delivered to treatment sites via a collagen sponge. A supraphysiological dose of the growth factor is used for clinical applications based on the observation that BMP2 is retained at the treatment site only for short periods of time in vivo, due in part to rapid diffusion away from the intended site. This makes it difficult to sustain concentrations of the protein necessary to get optimal therapeutic effect at the correct time, necessitating the loading of higher‐than‐normal amounts of the biologic to the scaffold in an attempt to provide sustained BMP2 delivery. These higher BMP2 doses increase cost and increase the risk of side effects, thereby necessitating a high concentration to ensure the intended biological effect [28, 29, 30]. Unfortunately, the burst release of BMP2 can cause off‐target effects such as heterotopic ossification and persistent soft tissue inflammation, leading to serious medical complications and further clinical intervention [31, 32].

We reasoned that the copper‐free click hydrogel system would provide an effective alternative to the collagen sponge due to its ability to conform to the parameters of the treatment site and degrade over time without causing tissue toxicity and release the payload in its bioactive form. There has been an extensive amount of research directed toward more effective BMP2 delivery vehicles. Hydrogels are one strategy pursued as a solution to this BMP2 delivery problem. Injectable hydrogels are formed from mutually reactive soluble precursors that react in situ to form insoluble networks with a defined geometry to contain and deliver biologics [33, 34]. The in situ formation gives these hydrogels the ability to freely conform on the macro‐scale to defect shape and localize protein delivery. What makes these hydrogels more ideal is their ability to sustain the delivery of biologics over a period of time, overcoming some of the issues around a traditional collagen scaffold. Despite these improvements, injectable hydrogels are not without their own issues. Principally, common hydrogel formation chemistries are too slow to encapsulate the biologic being delivered and share the same issue as the collagen scaffold: the molecule diffuses away from the intended site rapidly. Many hydrogels require some type of initiator to trigger the polymerization reaction, such as UV light or metal–ligand interaction. UV light has the issue of not being able to be used in deeper tissues without invasive surgery, as the components require exposure to the light source to polymerize; even when UV light is successfully introduced into tissues, it has a shallow depth of penetration due to endogenous chromophore attenuation [35] and is toxic to cells. Metal–ligand and other precursors can react with cellular components and be toxic to skeletal tissues. In addition, hydrogels tend to swell in vivo, causing mechanical strain on the underlying tissues. Thus, an injectable hydrogel that polymerizes rapidly in situ, does not require an outside initiator, does not generate heat, and exhibits only minimal swelling is highly desirable as it can encapsulate the biologic, preventing immediate diffusion and is more beneficial to patient safety.

The goal of this study was to examine if the Cu^2+^‐free click hydrogel system could serve as an improved alternative to clinical methods of BMP2 delivery. We initiated our analysis by establishing the systemic safety of the hydrogel formulation used as a delivery vehicle for anti‐VEGF antibody and topotecan (formula F1) [21] with respect to the health of peripheral organs and any immunogenicity. Potential cytotoxicity of the hydrogel degradation products was determined using three different cell lines in vitro. After modifying the formulation to facilitate the synthesis of the crosslinker (formula F2) [22, 23], BMP2 was incorporated into the hydrogel, and the kinetics of its release were determined. We assessed the bioactivity of the released BMP2 over time in vitro. Lastly, we compared the osteogenesis elicited by the BMP2 delivered via the hydrogel to that elicited by BMP2 delivered via collagen in an in vivo calvaria model [21, 24, 36, 37].

## 2 Materials and Methods

### 2.1 Hydrogel Material Preparation

Two formulations of the rapidly polymerizing hydrogel were used in this study. Formula F1 was used in the initial studies assessing safety in vitro and in vivo. In brief, PEG‐N3 was synthesized from azide‐functionalized and non‐functionalized PEG methacrylate monomers through reversible addition‐fragmentation chain transfer polymerization, resulting in tight control of azide functionality [38]. The generated polymer has a molecular weight of approximately 25 kDa with an average azide functionality per polymer of 13. The difunctionalized PEG‐DBCO crosslinker was synthesized by thiol‐Michel addition reaction between PEG‐dithiol and dibenzocyclooctyne maleimide (DBCO‐maleimide). The DBCO‐functionalized precursor forms an in situ crosslinked hydrogel with azide‐functionalized acylate polymer.

Formula F2 was used for BMP2 release studies and in vivo assessment of hydrogel + BMP2 effectiveness. F2 is a good laboratory practice (GLP) commercial scale‐up of the previous lab‐scale synthesis. The components of the hydrogel were synthesized according to our specifications at a commercial facility (Syngene International, Bengaluru, India), and shipped frozen to our laboratory. The components were reconstituted in sterile 1X PBS (ThermoFisher Scientific, Waltham, MA, USA) and stored at −80°C before use. The hydrogel was formed by mixing two parts of PEG‐DBCO (12.5%; w:v) and one part of PEG‐N3 (50%; w:v) using a dual‐syringe dispensing apparatus that dispenses the two solutions simultaneously at a 2:1 ratio. Recombinant human bone morphogenetic protein‐2 (rhBMP2) was purchased from R&D Systems.

### 2.2 In Vitro Hydrogel Safety Testing

#### 2.2.1 Cytotoxicity

Hydrogel cytotoxicity testing was done according to the standard methods indicated below. We used three different cell lines for these studies, all from ATCC: human MG63 osteoprogenitor cells, mouse MC3T3‐E1, clone 4 osteoblast‐like cells, and human MRC5 lung fibroblasts. Two parts F1 PEG‐DBCO crosslinker and 1 part PEG‐N3 were incubated on ice until mixing by pipetting—the hydrogel click reaction, and thus gelation, proceed extremely slowly at 4°C, facilitating handling. Media were incubated in Eppendorf tubes containing 4.69 mm diameter latex (positive control), high density polyethylene disks (negative control) or F1 hydrogel for 24 h.

All cells were cultured in 96 well tissue culture polystyrene (TCPS) plates at a density of 5000 cells per centimeter square in Dulbecco's modified Eagle medium (DMEM) containing 10% fetal bovine serum (FBS). At confluence, cells were treated for 24 h with media from the disks in the Eppendorf tubes or media from an empty Eppendorf tube. At that time, the culture media were replaced with fresh serum‐free DMEM. An MTT assay (Sigma‐Aldrich; M2128) was performed according to the manufacturer's protocol.

#### 2.2.2 Hydrogel + BMP2 Analysis

##### 2.2.2.1 BMP2 Release Kinetics and Activity In Vitro

All in vitro experiments were performed under aseptic conditions. Aqueous stock solutions of F2 PEG‐DBCO (12.5%; w:v) crosslinker and PEG‐N3 copolymer (50%; w:v) were prepared by vortexing and sonicating the polymers in PBS at room temperature. Both components were filtered using a 50 mL centrifuge tube filter system containing a 0.22 μm polyethersulfone filter (Celltreat, Pepperell, MA).

BMP2 release kinetics: Hydrogels were formed using a 12.5% (w/v) PEG‐DBCO crosslinker to PEG‐N3 (2:1 v/v). Prior to formation, the crosslinker was seeded with BMP2. Crosslinker and PEG‐N3 were mixed via pipetting and 16 μL aliquots, containing 100 ng BMP2 each, were allowed to crosslink in the bottom of 0.65 mL Eppendorf centrifuge tubes. 100 μL of full media (DMEM with 10% FBS, 0.5% penicillin/streptomycin) was added to each sample, and at each time point, all 100 μL of media were removed for quantification and replaced with fresh media. Each sample contained 16.67 ng of BMP2. Results were quantified using a Human BMP‐2 ELISA (R&D Systems DY 355‐05 Human BMP‐2 Duoset ELISA).

BMP2 bioactivity: The biological activity of BMP2 delivered from the hydrogel was performed using MG63 cell cultures. MG63 cells were grown in DMEM with 10% FBS. Gels containing 100 ng rhBMP2 were prepared in Eppendorf tubes as described above and were incubated in 0.5 mL DMEM with 10% FBS at 37°C for 2 days. The media were collected and any remaining hydrogel was separated by centrifugation. The supernatant media were diluted 1:6 with fresh media, and 0.5 mL of conditioned media, containing approximately 10 ng of BMP2, was added to 6 wells of a 24‐well TCPS culture plate when the MG63 cells were at 80% confluence, or DMEM with 10% FBS +10 ng rhBMP2, or DMEM with 10% FBS. After 48 h, cells were harvested and tested for proliferation by measuring the total amount of DNA in cell layer lysates and markers of osteoblastic differentiation in the conditioned media, including osteocalcin, osteopontin, and osteoprotegerin [22].

In a separate set of experiments, a co‐culture system was used. MG63 cells were cultured on 24‐well TCPS plates with well inserts (Thermo Fisher Scientific; 141 002). Hydrogels with or without 10 ng rhBMP2 (two parts PEG‐DBCO and 1 part PEG‐N3) were cast on the inserts and lowered into each well containing 500 μL of culture media when the cells were at 80% confluence; cells were harvested 48 h later. Test groups consisted of: hydrogel alone; hydrogel + BMP2; Empty insert; Empty insert plus addition of 20 ng/mL rhBMP2 to the media.

### 2.3 In Vivo Safety Testing

All animal studies were performed with the approval of the Institutional Animal Care and Use Committee at Virginia Commonwealth University in accordance with the Guide for the Care and Use of Laboratory Animals 8th Edition [39].

#### 2.3.1 Intracutaneous Injection of Hydrogel Extract in Rabbit

Extraction fluid was prepared following *ASTM Standard F619‐03: Standard Practice for Extraction of Medical Plastics* [40]. Two parts of F1 PEG‐DBCO (12.5%; w:v) click hydrogel crosslinker were combined with 1 part PEG‐N3 (50%; w:v) to form a 1.6 g hydrogel. This was combined with 5 mL 0.9% NaCl in a sterilized borosilicate glass container and placed in a water bath at 37°C, capable of agitation. The container was observed for signs of mixing and removed from the water bath, then shaken vigorously for 30 s before decanting the extract liquid into a sterile container.

All immunogenicity studies were performed using F1 hydrogel extract under an Institutional Animal Care and Use Committee at Virginia Commonwealth University and followed NIH guidelines. Two male New Zealand White rabbits were prepared for injection by shaving a large area of the back on both sides of the spinal column providing for a sufficient test area. Loose hair was removed by means of a vacuum, and the skin was sterilized with alcohol swabs. Extract liquid was agitated prior to withdrawal of each injection dose. Rabbits were injected intracutaneously with 0.2 mL of hydrogel extract at five sites on the same side of the animal. 0.2 mL of 0.9% NaCl solution was injected at five sites on the opposite side of the back. Thus, each animal had 10 injections, five with saline as control and five with the hydrogel extract. Two animals were tested, resulting in 10 sites that had the treatment and 10 sites that had saline. The injection sites were examined at 24, 48, and 72 h for gross evidence of tissue reaction, such as erythema, edema, or necrosis. Scoring was based on tables from the *ASTM Standard F749‐13*: *Standard Practice for Evaluating Material Extracts by Intracutaneous Injection in the Rabbit* [41].

#### 2.3.2 Guinea Pig Maximization Test

Extraction fluid was prepared as above, following *ASTM Standard F619‐03: Standard Practice for Extraction of Medical Plastics* [40]. Test samples were prepared for intradermal injection by combining 0.05 mL Freund's complete adjuvant with 0.05 mL 0.9% NaCl, 0.05 mL extract. Mixtures were homogenized by continuous vortex for 5 min. Twenty‐seven male Guinea pigs were used for this study. Animals were shaved at the shoulder region exposing a 4 × 6 cm area and three injection sites were chosen at least 1.5 cm apart. One week following injections the area was re‐shaved and treated with 10% sodium lauryl sulfate (SLS) in petroleum jelly 24 h prior to applying test patches. Test samples, including pbs as a negative control, mercaptobenzothiazole (MBT) as a positive control, and hydrogel extract in 0.9% NaCl or sesame oil, were mixed with petroleum jelly and applied to a 2 × 4 cm filter paper until saturated. The filter paper was then placed on the injection site and secured with occlusive surgical tape and an elastic bandage for 48 h. Two weeks later a 5 × 5 cm area was shaved on the animal's flank and filter paper saturated in the test agent was applied to the animal in the same manner for 24 h. After removing the patches, test sites were examined at 1 h, 24 h, and 48 h for signs of erythema and edema. Scoring was done in accordance with *ASTM Standard F720‐81: Standard Practice for Testing Guinea Pigs for Contact Allergens: Guinea Pig Maximization Test* [42] and the allergenicity of the hydrogel was determined.

#### 2.3.3 Systemic Pathology Following Treatment of Craniotomy Defects

Six 45‐day‐old C57BL6 mice were purchased (Jackson Labs, Bar Harbor, ME) and allowed access to ad libitum food and water in a light‐ and temperature‐controlled environment for a week prior to surgery. On the day of surgery, mice were anesthetized with 2.5% isoflurane in 400 mL/min oxygen. The hair over the posterior frontal suture of the skull was removed and sterilized by three alternations of alcohol and chlorhexidine solution. A 1 mm mid‐sagittal incision was made 5 mm posterior to the eyes. The periosteum overlying the cranium was elevated and removed from the underlying bone. A 5 mm critical size bone defect was made in the middle of the right parietal bone for all animals, taking care not to damage the underlying dura. At this step, the defects were left empty (*N* = 3) or the F1 hydrogel was placed in the defect by simultaneously pipetting the 12.5% PEG‐DBCO (w.v) crosslinker and PEG‐N3 (2:1 v.v) so that each defect was filled (*N* = 3). The hydrogel was allowed to crosslink for 45 s to 1 min. Polymerization was verified with a blunt 25G needle. The overlying skin was reapproximated and closed with 5–0 nylon suture in an interrupted technique.

Brain, kidney, liver, and spleen were harvested from mice with empty defects or defects treated with hydrogel alone and were fixed in 10% neutral buffered formalin, changing the solution after 24 h. The samples were dehydrated with ethanol and embedded in paraffin. Sections 7 μm in thickness were made and stained with H&E using standard protocols. The samples were imaged at 10× magnification and analyzed for any pathology or hydrogel particles.

### 2.4 In Vivo Effectiveness of rhBMP2 Delivery by F2 Hydrogels

A power analysis was performed based on our published studies using this model to test the effectiveness of the F1 hydrogel to deliver biologics [20, 21]. Forty 45‐day‐old C57BL/6 mice were purchased (Jackson Labs, Bar Harbor, ME) and allowed access to ad libitum food and water in a light‐ and temperature‐controlled environment for a week prior to surgery. On the day of surgery, mice were anesthetized with 2.5% isoflurane in 400 mL/min oxygen. The surgical protocol described above was followed. Mice were divided randomly into five experimental groups of eight animals each: empty defect; collagen scaffold seeded with 1 μg rhBMP2 (as per the clinical guide for using BMP2 with collagen); hydrogel alone; and hydrogel with 1 μg rhBMP2. The hydrogel was allowed to crosslink for 45 s to 1 min. Polymerization was verified with a blunt 25G needle before closing the skin with 5–0 nylon sutures. An additional group of four mice was immediately euthanized following the creation of the defect in order to characterize the parameters of the initial craniotomy. Following surgery, mice were given postoperative analgesia for the first 72 h and access to ground, moistened food and water to encourage weight gain. At 28 days postsurgery, animals were euthanized.

For this study, we used recombinant human/mouse/rat BMP2 (R&D Systems 355‐BM‐010). 10 μg BMP2 was reconstituted with 106.6 μL of crosslinker. 10.66 μL of crosslinker was combined with 5.33 μL of PEG copolymer to yield 16 μL total volume hydrogel +/− 1 μg of BMP2 upon application to the 5 mm diameter cranial defect. We used a 5 mm internal diameter biopsy punch (Miltex Catalogue 9534123‐EA) to punch 5 mm diameter sponges from resorbable collagen tape (Pearson Dental A23‐0150). BMP2 was dissolved in PBS and pipetted in 10 μL aliquots containing 1 μg each to the collagen sponge that was placed in the 5 mm diameter cranial defect.

#### 2.4.1 Vasculogenesis

To assess vasculogenesis, mice were perfused with a radio‐opaque silicone‐based contrast agent to visualize angiogenesis in and around the defect. Mice were euthanized by exsanguination while in a deep plane of anesthesia. Briefly, mice were anesthetized with 4% isoflurane in 400 mL/min oxygen and positioned supine with their limbs restrained. An incision was made down the centerline of the sternum, and the ribcage was removed to expose the heart. A catheter was placed in the apex of the left ventricle, and whole blood was collected for serum isolation. The inferior vena cava was ligated to allow for blood and other solutions to clear the vasculature. A peristaltic pump attached to the ventricular catheter delivered heparinized PBS (50 U/mL), followed by 10% neutral buffered formalin (NBF) perfusion throughout the mouse. The descending aorta was then clamped in order to localize the Microfil solution (Microfil, Flow‐Tech Inc., Carver, MA) to the head and neck region. Following perfusion, Microfil was allowed to cure for 90 min at room temperature. The skull was then placed in 10% NBF for 48 h prior to microCT imaging and histologic preparation and analysis.

#### 2.4.2 MicroCT Analysis

Bone healing and angiogenesis in and around the defect were assessed. Calcified specimens were scanned by microCT to assess bone formation. Skulls were then decalcified using Decal Decalcifier (StatLab 1211‐1 GAL) according to the manufacturer's instructions until complete decalcification was achieved. A second scan was then done in order to visualize the vasculature alone. All scans were performed on a Bruker Skyscan 1173 table‐top microCT at a resolution of 1120 × 1120 pixels, with an image pixel size of 15.10 μm, and scanning energy of 87 kV and 57 μA with no filter, an exposure time of 250 ms, and frame averaging of five images per 0.2° rotation step. A standard Feldkamp reconstruction was performed on a subset of samples using NRecon Software 1.7.4.4 (Kontich, Belgium) with a Gaussian smoothing kernel of zero and a beam hardening correction of 20%. The calcified and decalcified scans were then aligned manually. The defect area was isolated, creating a volume of interest, and the radio‐opaque tissue in and around the defect was analyzed using CTAn software 1.18.4.0. The defect volume was quantified in the calcified scans, and the amount of radio‐opaque material within the boundaries of the defect, the ectopic radio‐opaque material outside of the boundaries of the defect, and the total amount of radio‐opaque material (defect+ectopic) were quantified as %Bone Volume over Total Volume. The total volume of Microfil‐perfused vessels within the volume of interest was quantified, as well as the volume of vessels contained within the margins of the calvarial defect. The % of the defect occupied by blood vessels was also quantified. The mean total cross‐sectional vessel area across all microCT 2‐dimensional slices within the margins of the defect, and the average number of vessels per slice were also analyzed.

#### 2.4.3 Histomorphometric Analysis

Histological assessment was performed by hematoxylin and eosin (H&E) staining of decalcified 7 μm thick axial sections in the middle of the defect and analyzed by light microscopy, as described below. After imaging with microCT, the brain was removed, taking care not to damage the defect. The samples were fixed in 10% neutral buffered formalin, changing the solution after 24 h. The skulls were decalcified by immersion in Decal Decalcifier for a period of 72 h. Complete decalcification was verified by slicing through the rostrum. The center of the defect was visualized under 4× magnification, and a coronal cut was made through the center of the defect. The samples were dehydrated with ethanol and embedded in paraffin. Sections 7 μm in thickness were made and stained with H&E using standard protocols. The samples were imaged at 10× magnification. The average defect width and area of bone formation in and around the defect were calculated (Fiji; ImageJ).

### 2.5 Statistical Analysis

All data are represented as the mean ± standard error of the mean. The sample size for all in vivo and in vitro experiments was determined by a prospective power analysis based on previously reported data. All cell culture experiments were performed with six independent cultures (*n* = 6) and repeated at least two times. All in vivo experiments were performed with at least six mice per group (*n* = 6). For all in vitro and in vivo experiments, a one‐way ANOVA was performed, and significance among groups was determined by a multiple comparison test with Tukey adjustments. Statistical significance for all experiments was declared when the *p*‐value was less than 0.05.

## 3 Results

### 3.1 Hydrogel Safety Testing In Vitro

**FIGURE 1:**
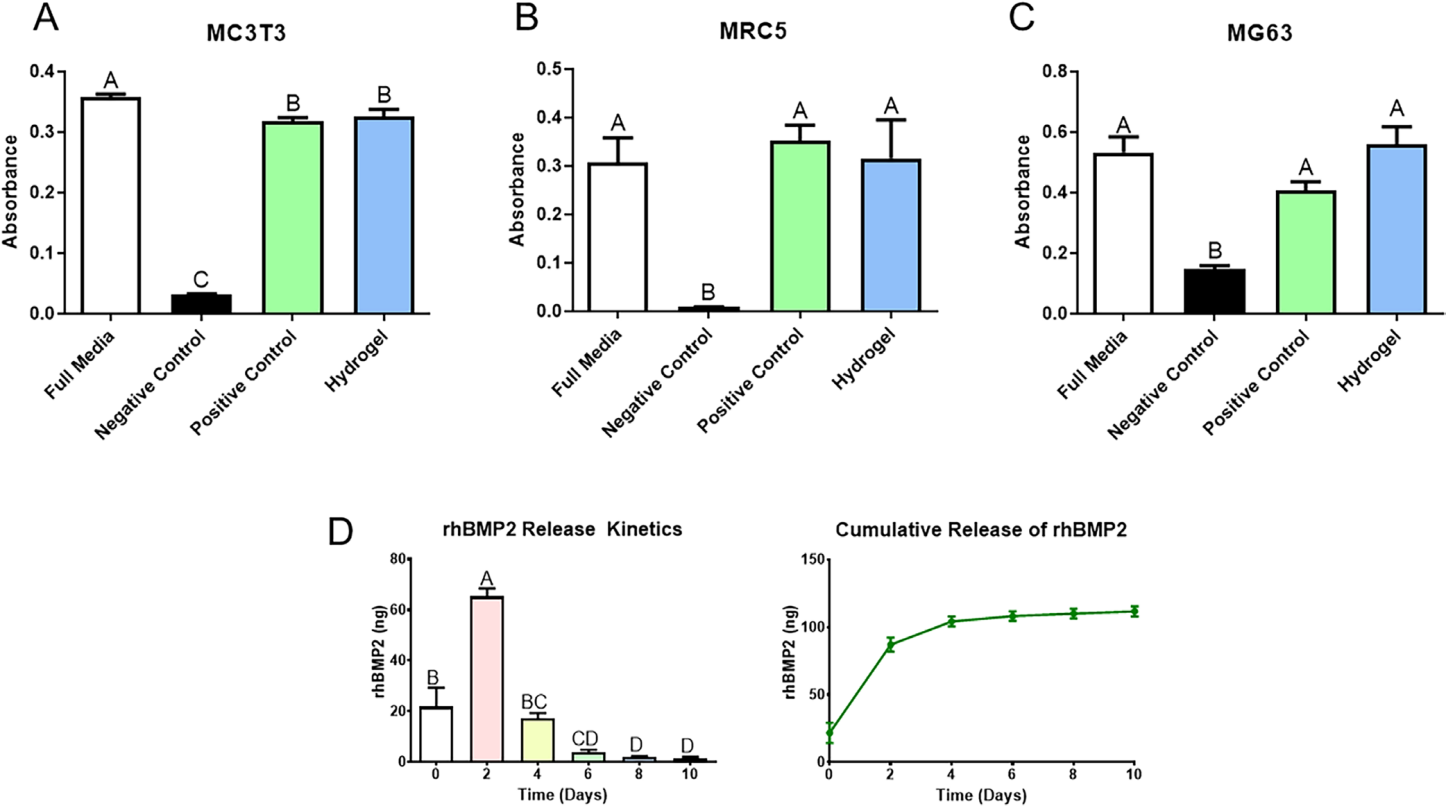
Characterization of hydrogel. Hydrogel safety testing. Results of MTT assays to test cytotoxicity of the hydrogel. Media were untreated or incubated on a polyethylene disc, latex disc, or the hydrogel and then used to treat cells. MTT absorbance assay was used to quantify cell death (*n* = 6). Cell lines examined include (A) MC3T3 mouse osteoblasts, (B) MRC5 human fibroblasts, (C) MG63 human osteoblasts. (D) The release of rhBMP2 from the hydrogel was assessed over 10 days. Hydrogels were incubated in 100 μL of media and aliquots were taken at each time point (*n* = 6). The graphs report the amount released per day and the cumulative release respectively. Differences between groups not sharing a letter are statistically significant (*p* ≤ 0.05).

The hydrogel was not cytotoxic when tested in vitro. MC3T3 cells treated with media that were incubated on high‐density polyethylene disks (negative control) exhibited a significant reduction in MTT compared to media from empty Eppendorf tubes. The media incubated with latex or hydrogel had no effect on the MTT level compared to the control samples (Figure 1A). Similar results were observed with MRC5 cells (Figure 1B) and MG63 cells (Figure 1C).

### 3.2 BMP2 Release Kinetics

BMP2 exhibited sustained release from the hydrogel, following an initial burst release between days 0 and 2, with a large burst occurring on day 2 (Figure 1D, left graph). By the end of day 2, 87% of the BMP2 had been released from the hydrogel. The remaining BMP2 was then slowly released over the following 8 days, with a sustained gradual decrease through 10 days until no BMP2 remained in the hydrogels.

### 3.3 Bioactivity of BMP2 Released From Hydrogel

**FIGURE 2:**
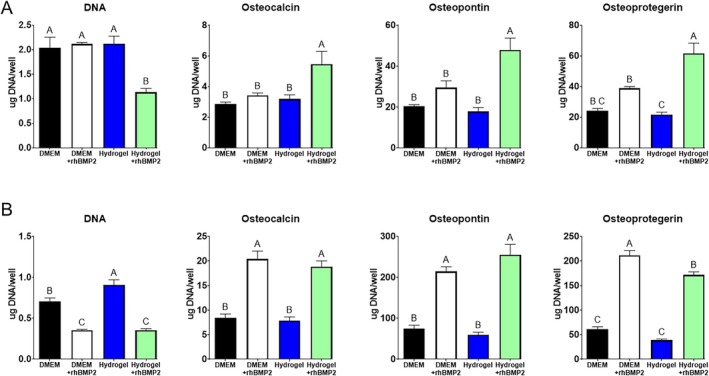
Validation of biologic activity of rhBMP2 following hydrogel release. (A) Conditioned media experiment to confirm biological activity of rhBMP2 following release. Quantified DNA and osteoblast differentiation markers (*n* = 6). Results of protein ELISAs are normalized to DNA content. (B) Coculture experiment to show rhBMP2 was active immediately following release of hydrogel. Hydrogels were suspended above cell monolayer via well insert. Quantified DNA and osteoblast differentiation markers (*n* = 6) Differences between groups not sharing a letter are statistically significant (*p* ≤ 0.05).

Two experiments were performed to ensure that the biological effect of the BMP2 was preserved after being released from the hydrogel. First, conditioned media that had been exposed to the hydrogel containing BMP2 was used to treat osteoblast progenitor MG63 cells (Figure 2A). The DNA content of MG63 cell cultures treated with BMP2 released from the hydrogel was significantly reduced when compared to media from control cultures or empty hydrogel, or conditioned media from cells treated with BMP2. MG63 cells treated with rhBMP2 released from the hydrogel exhibited increased production of osteocalcin, osteopontin, and osteoprotegerin compared to all other groups measured. No difference was found between cells treated with BMP2 or empty gel compared to control when osteocalcin or osteopontin were measured. When osteoprotegerin was measured, the addition of BMP2 increased its level compared to empty hydrogel but not control.

A transwell culture system was used to further confirm the bioactivity of the BMP2 released from the hydrogel. Hydrogels containing BMP2 were placed on a porous membrane suspended above the well plate surface and allowed to release BMP2 into the media over 48 h (Figure 2B). BMP2 released from the hydrogel had a similar effect to the positive control, which had BMP2 supplemented in the media. Both test groups showed a decrease in DNA content and an increase in differentiation as exhibited by heightened amounts of osteopontin, osteocalcin, and osteoprotegerin compared to control or empty hydrogel, although with osteoprotegerin, the level was significantly reduced in hydrogel + BMP2 than in the addition of BMP2.

### 3.4 Hydrogel Safety Testing In Vivo

In the rabbit subcutaneous sensitization test, the injection sites exhibited only minor signs of erythema (four of 10) and edema (two of 10) 24 h after injection. After 48 and 72 h, there were no signs of edema or erythema on any of the injection sites (Table 1). The Guinea Pig Sensitization Test did not show signs of allergic response to the hydrogel extract in PBS or sesame oil. Tissue was similar to PBS control. In contrast, mercaptobenzothiazole (MBT) caused sensitivity in all the samples (Table 2).

**TABLE 1:** Rabbit intracutaneous sensitization test.

Animal: Rabbit 1/Rabbit 2		Time: 24 h		Animal: Rabbit 1/Rabbit 2		Time: 48 h		Animal: Rabbit 1/Rabbit 2		Time: 72 h	
Site	Treatment	Edema	Erythema	Site	Treatment	Edema	Erythema	Site	Treatment	Edema	Erythema
1	Hydrogel extract	0/1	0/1	1	Hydrogel extract	0/0	0/0	1	Hydrogel extract	0/0	0/0
2	Hydrogel extract	0/1	0/1	2	Hydrogel extract	0/0	0/0	2	Hydrogel extract	0/0	0/0
3	Hydrogel extract	0/0	0/0	3	Hydrogel extract	0/0	0/0	3	Hydrogel extract	0/0	0/0
4	Hydrogel extract	0/0	0/0	4	Hydrogel extract	0/0	0/0	4	Hydrogel extract	0/0	0/0
5	Hydrogel extract	0/0	1/1	5	Hydrogel extract	0/0	0/0	5	Hydrogel extract	0/0	0/0
6	Blank	0/0	0/0	6	Blank	0/0	0/0	6	Blank	0/0	0/0
7	Blank	0/0	0/0	7	Blank	0/0	0/0	7	Blank	0/0	0/0
8	Blank	0/0	0/0	8	Blank	0/0	0/0	8	Blank	0/0	0/0
9	Blank	0/0	0/0	9	Blank	0/0	0/0	9	Blank	0/0	0/0
10	Blank	0/0	0/0	10	Blank	0/0	0/0	10	Blank	0/0	0/0

**TABLE 2:** Guinea pig sensitization test.

Groups results		
	Rating of sensitization response	
	% Animals sensitized	Classification
PBS negative control	0/5 = 0%	—
Positive control—Mercaptobenzothiazole (MBT)	4/4 = 100%	Extreme
Hydrogel extract in NaCL (Test condition 1)	0/10 = 0%	Not different than negative control
Hydrogel extract in Sesame oil (Test condition 2)	0/8 = 0%	Not different than negative control

### 3.5 In Vivo Calvarial Defect Study

**FIGURE 3:**
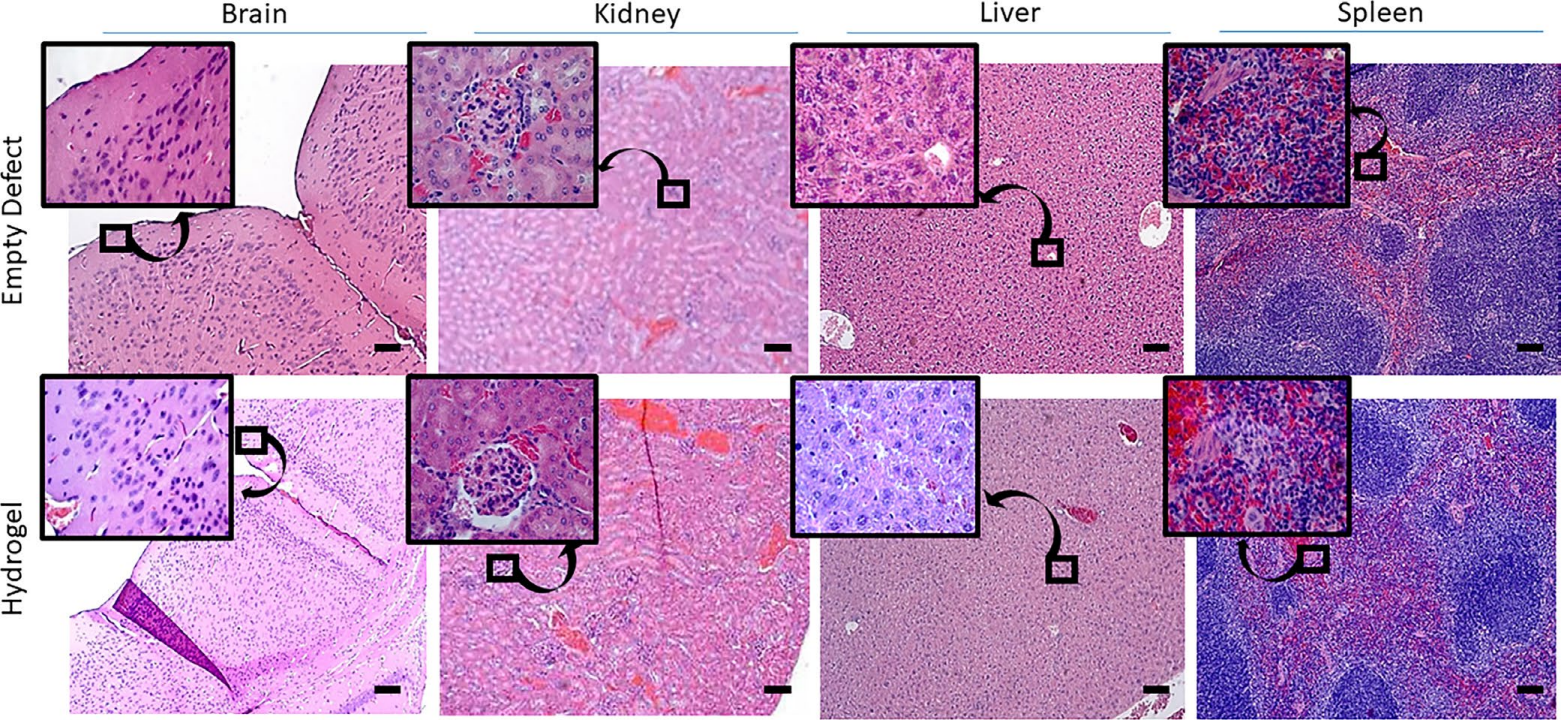
Hydrogel safety testing. Histology sections of major organs including the brain, kidney, liver, and spleen to show that hydrogel particles do not impact organ function. Sections taken at 10×, inset at 63×. Scale bar = 100 μm. Differences between groups not sharing a letter are statistically significant (*p* ≤ 0.05).

Mice were euthanized 4 weeks after implantation of the hydrogel alone or hydrogel + BMP2 versus collagen alone or collagen + BMP2 to assess bone formation. Major organs were collected from mice receiving hydrogel implants alone. There was no evidence of systemic toxicity in major organs collected from animals that had critical‐size calvarial defects treated with hydrogel alone. No gross morphological changes were noted. Moreover, histology sections did not demonstrate the presence of any hydrogel particulate that may have been released as the hydrogel degraded in the body. There was no evidence of any pathology or inflammation (Figure 3).

**FIGURE 4:**
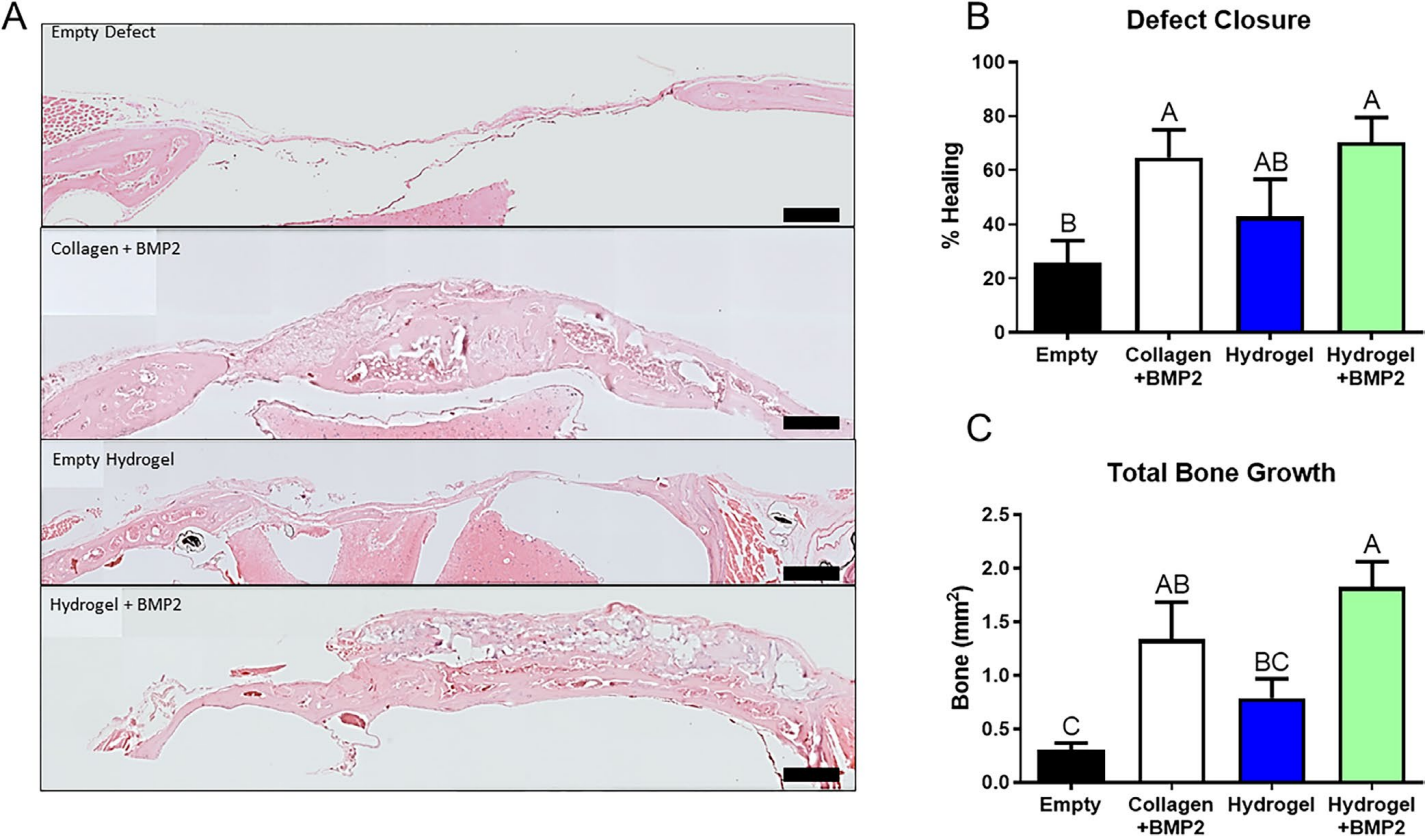
Histology. (A) Representative histologic hematoxylin and eosin‐stained sections taken from the middle of the defect based on the sagittal plane. Sections imaged at 10×. (B): Quantification of defect closure and total bone growth using Fiji. Defect closure represents length from new bone growth to the edge of the defect. (C) Total bone growth is a measurement of the area of new bone growth shown in the histology section. Scale bar = 400 μm. Differences between groups not sharing a letter are statistically significant (*p* ≤ 0.05).

Histomorphometric analysis was performed using ImageJ software of H&E‐stained sections (Figure 4A). Defect closure was based on the length measured from the margin of the defect to new bone growth and presented as percent healing (Figure 4B). Both the collagen + BMP2 and hydrogel + BMP2 sites had significant defect closure compared to the empty defect. Hydrogel + BMP2 defects had similar closure to the other groups. Total bone growth was measured as the area of new bone growth shown in the section, both inside and outside the margins of the defect (Figure 4C). The area of new bone formation for the hydrogel+BMP2 treated defects was significant compared to the empty hydrogel and empty defect. Collagen+BMP2 sites showed a trend of increased bone formation, which was significant compared to the empty defect, but not when compared to the empty hydrogel.

**FIGURE 5:**
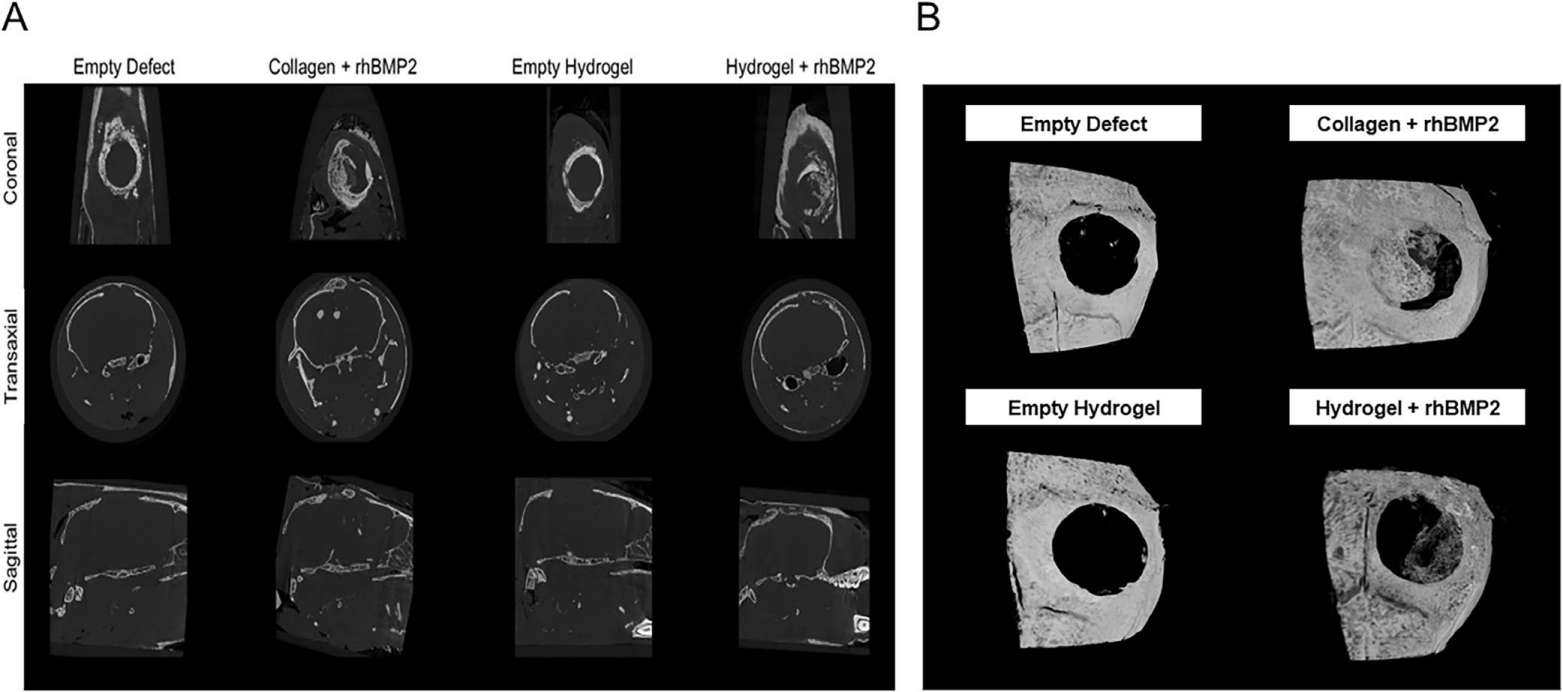
Bone regeneration. (A) Representative 2‐dimensional μCT mid‐defect images of samples before decalcification showing the amount of new bone regenerating in the 5 mm critical‐sized defect. Coronal (top), Transaxial (middle), and Sagittal (bottom) planes of each specimen (*n* = 6). (B): Top‐down 3‐dimensional representative images of each group.

MicroCT analysis confirmed these histologic findings (Figure 5). The tissue volume shows that the region of interest selected around the defect was consistent across all groups that received a treatment. Analysis was performed using Bruker CTan, and bone inside the defect was measured by drawing a region of interest within the margins of the defect. The amount of bone inside the defect was normalized to tissue volume to examine how effectively the defect closed over 4 weeks. The microCT cross section (Figure 5A) and 3D reconstruction (Figure 5B) show production of new bone in the defects treated with collagen+rhBMP2 and hydrogel+rhBMP2. No bone regeneration was found in the empty defect or empty hydrogel groups. More bone was found in the collagen+rhBMP2 sites than in hydrogel+rhBMP2 groups. However, significantly more ectopic bone was found in the collagen+rhBMP2 treated sites (Figure 7A).

**FIGURE 6:**
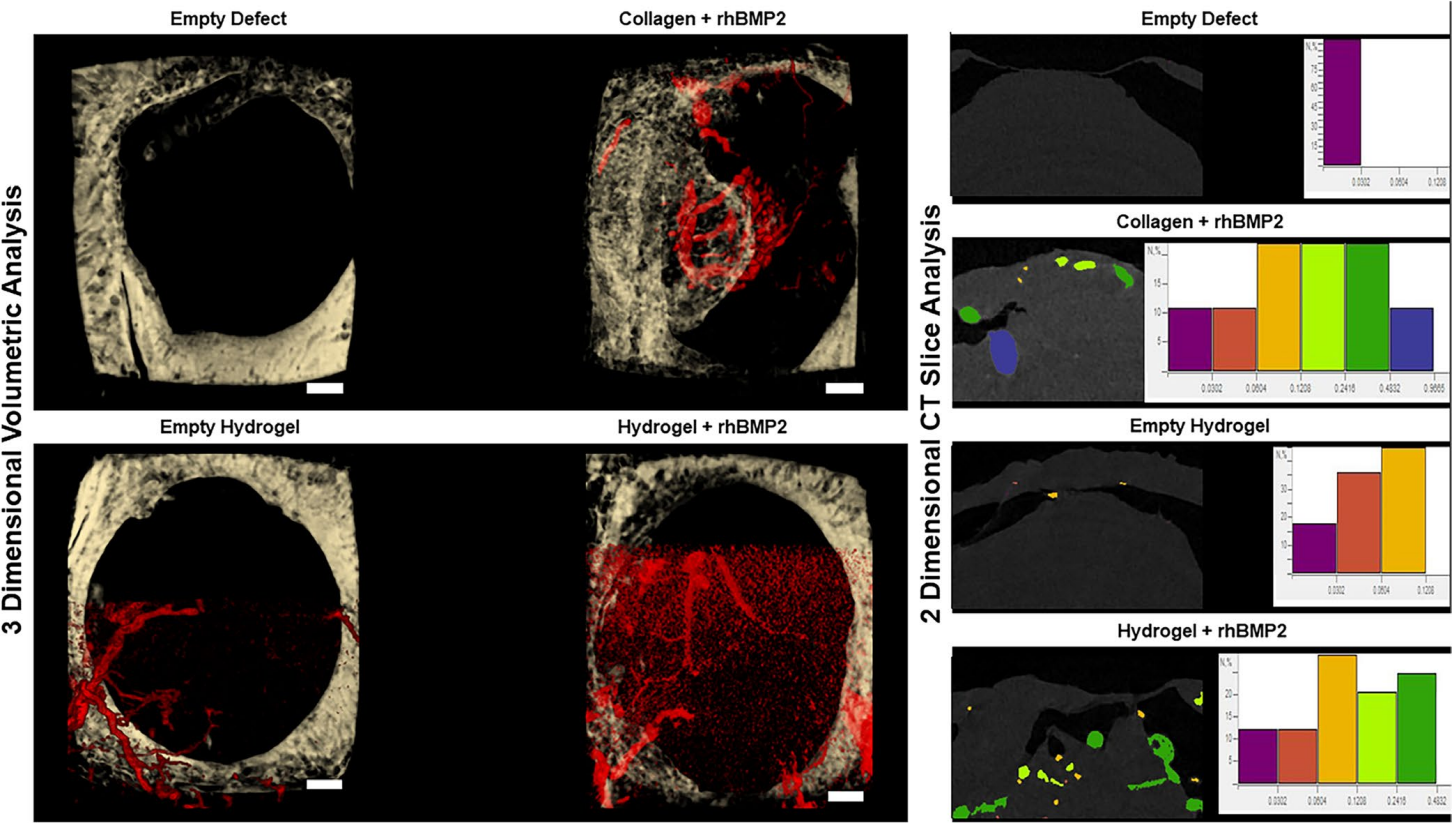
Angiogenesis. (Left): 3‐dimensional top‐down microCT overlay of the mineralized defect (white) and the decalcified image showing the amount of new blood vessels (red) within each defect. (Right): Representative 2‐dimensional mid‐defect slice of decalcified scan. Histograms show the size distribution of the vessels within the mid‐defect delineated by color. Y‐axis is number of vessels by %. X‐axis is the diameter of the longest axis (mm). Scale bar = 500 μm.

The presence of hydrogel within the defect led to enhanced vascularization of the site (Figure 6). 3D reconstructions of the defects after decalcification showed Microfil penetrating into the defects treated with hydrogel alone. Microfil was present in the sites treated with collagen + rhBMP2 and extending into the non‐bony area of the defect, but this was only on the edges of the new bone. In contrast, Microfil was present throughout the defect sites treated with hydrogel + rhBMP2. Analysis of blood vessel diameter in 2‐dimensional CT slices indicated that blood vessel formation preceded bone formation. The vessels in the collagen + rhBMP2 sites were uniform in size. In sites treated with hydrogel alone, the vessels were smaller in diameter, whereas in sites treated with hydrogel + rhBMP2, there was a greater distribution of smaller diameter vessels.

**FIGURE 7:**
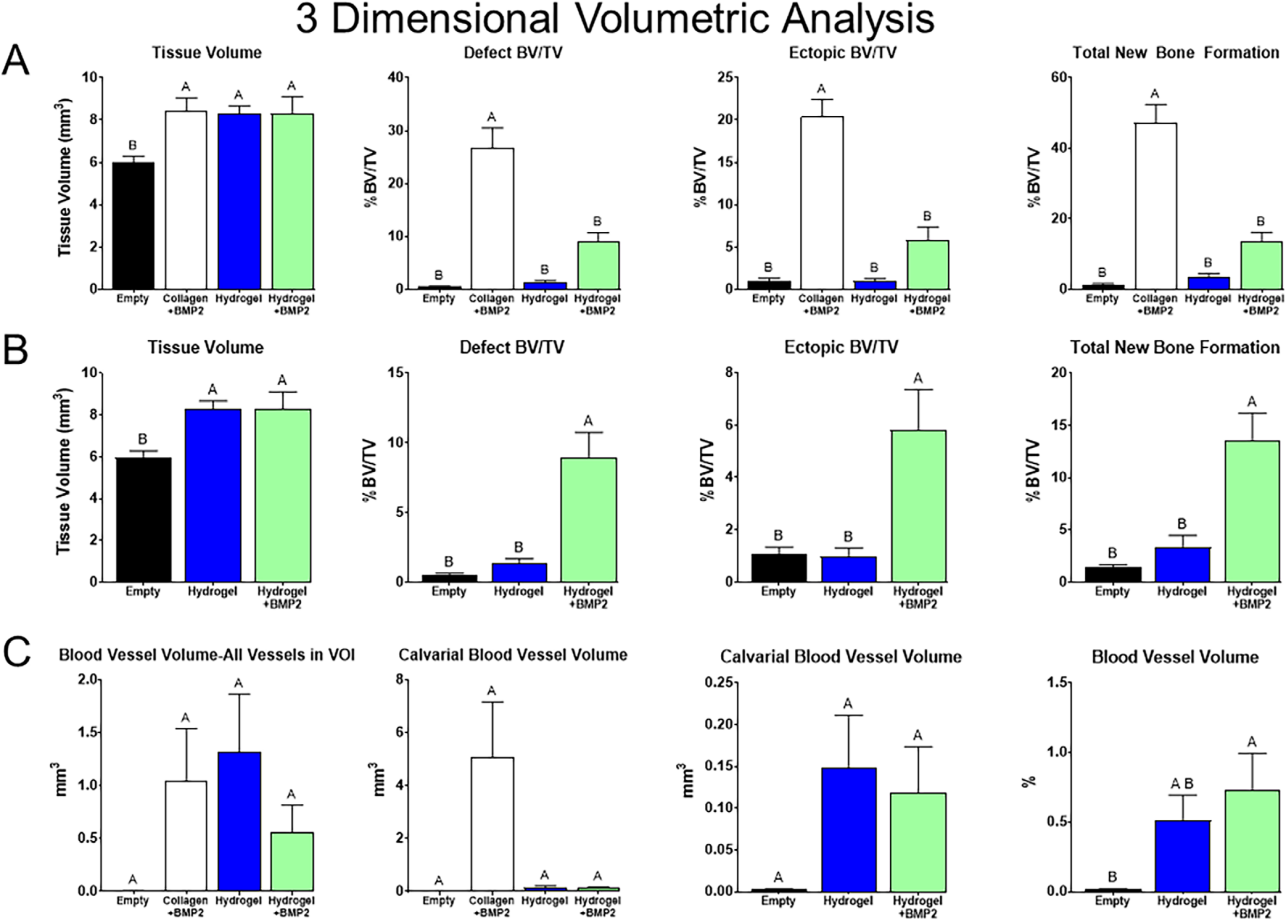
Quantification of bone and blood vessels. Histomorphometrics of μCT images from in vivo study showing from left to right‐total volume of the defect, total radio‐opaque tissue within the margins of the defect, ectopic radio‐opaque tissue outside the margins of the defect, and total radio‐opaque tissue (A) including collagen + rhBMP2 group and (B) excluding collagen + rhBMP2. (C) Quantification of blood vessels in the 3‐dimensional volume of interest. Graphs show from left to right‐all blood vessels within the volume of interest (mm^3^), volume of blood vessels confined to the calvaria (mm^3^) including collagen + rhBMP2, and excluding collagen + rhBMP2, blood vessel volume normalized to the defect volume. Differences between groups not sharing a letter are statistically significant (*p* ≤ 0.05).

Quantitative analysis of the microCTs made before decalcification showed that there was significantly more tissue in the defect in all treatment groups compared to empty defects, with no difference between the treatment groups (Figure 7A, left graph). Examination of the defect BV/TV (Figure 7A, left middle graph), ectopic BV/TV (Figure 7A, right middle graph), and total new bone (Figure 7A, right graph) shows that the addition of rhBMP2 to collagen significantly increased bone formation compared to all the other groups, with no difference between them. When the statistical analysis was made without collagen + BMP2, the stimulatory effect of BMP2 on bone formation became more apparent (Figure 7B). The analysis of bone formation between the empty defect, hydrogel alone, and hydrogel with BMP2 indicates a significant increase in bone formation in the hydrogel+ rhBMP2 sites compared to the other groups.

For these analyses, ectopic bone was measured as any bone growth outside the defect not consistent with the regular morphology of the parietal bone. The hydrogel+rhBMP2 treated sites showed the presence of bone growth outside the margins of the defect, but it was not significant compared to the control (Figure 7A). Evidence of this can be seen in the cross‐sectional views of the defect taken from the middle of the defect based on the sagittal plane (Figure 5A, right). The collagen+rhBMP2‐treated sites had a significant amount of bone growth outside the defect (Figure 7A, Figure 5A, left middle image). A majority of the animals had a mass of bone protruding outside the defect as shown in the cross section. Evidence of bone growth can be seen in both the hydrogel+rhBMP2 and collagen+rhBMP2 treated defects, while the empty hydrogel and empty defect show only thin lines of connective tissue spanning the defect (Figure 7B).

**FIGURE 8:**
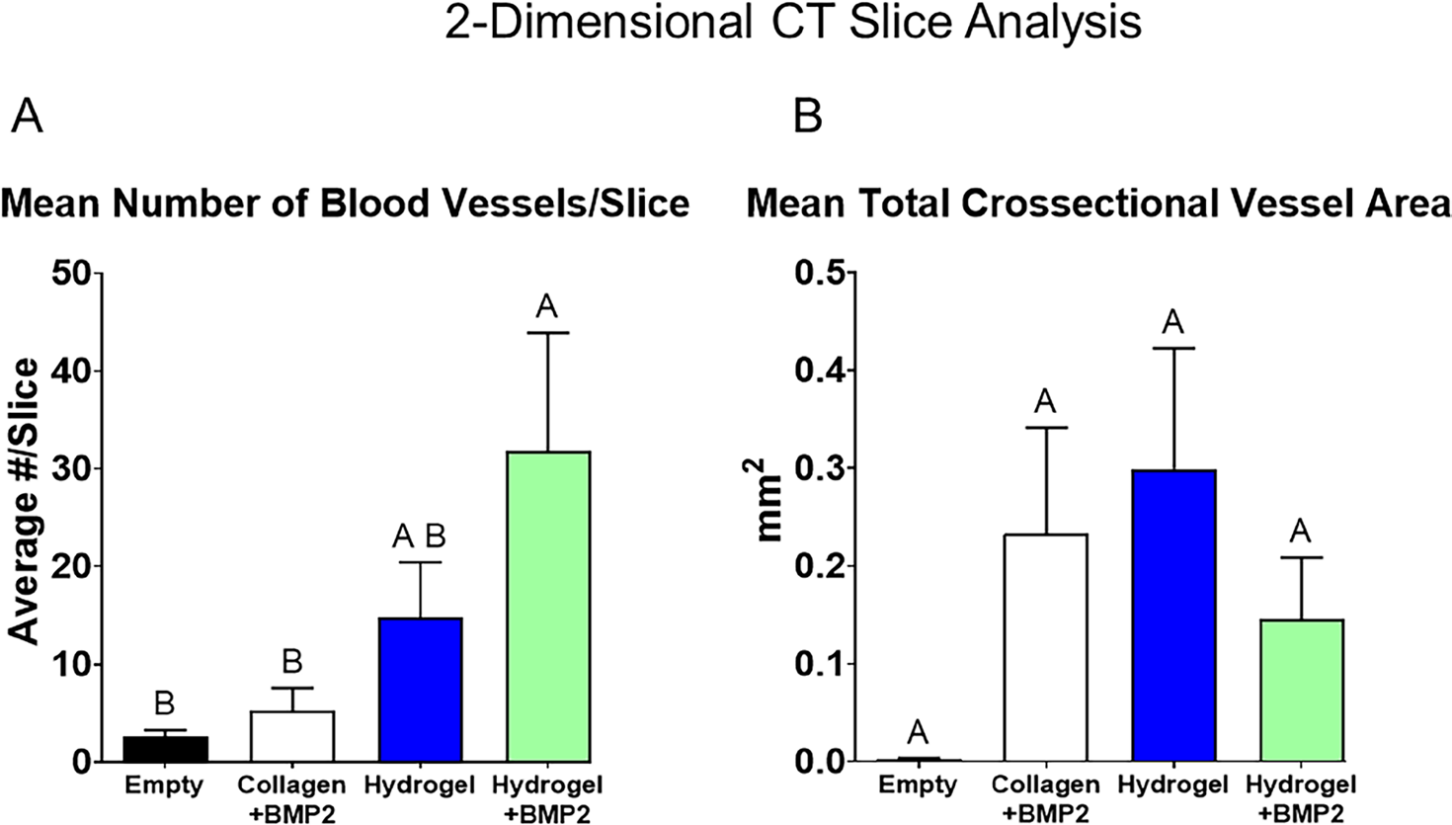
Blood vessel analysis. Histomorphometrics of μCT images from in vivo study showing 2‐dimensional blood vessel analysis of the middle of the defect. Data are the mean total cross‐sectional vessel area in mm^2^ (A) and the mean number of blood vessels per slice throughout the defect (B). Differences between groups not sharing a letter are statistically significant (*p* ≤ 0.05).

Angiogenesis in the samples was quantified. The total volume of all vessels in the volume of interest, as well as within the boundaries of the defect with and without collagen+BMP2, was equivalent across all groups (Figure 7C, left, left middle, right middle graphs). When examined as a percent of the total defect volume, hydrogel + BMP2 was significantly more compared to empty, but not compared to hydrogel, while hydrogel was equivalent to empty (Figure 7C, right graph). When analyzing angiogenesis per microCT slice across the defect, there was no difference in total cross‐sectional vessel area between the groups (Figure 8A). Hydrogel+BMP2 had a significantly higher average number of vessels per slice compared to empty and collagen+BMP2, while hydrogel was equivalent to all other groups (Figure 8B).

## 4 Discussion

Our results demonstrate that the click hydrogel described in this study is a viable method of delivery for rhBMP2 when used as a therapeutic for bone defect healing. While the hydrogel provided a burst release of BMP2 over the first 48 h after injection, it continued to release protein over the course of 10 days. This release profile is similar to heparin microparticles (HMPs), which exhibit an initial burst release followed by a gradual release with a retention of less than 5% of the initial dose after 2 weeks [30]. In contrast, rhBMP2 release from collagen occurs rapidly and is not sustained [43] 70% of the BMP2 is released from collagen within 10 h [44].

Moreover, rhBMP2 released from the hydrogel retained its biological activity in vitro. The increased production of osteogenic markers indicates the hydrogel exerted no deleterious effects on the encapsulated rhBMP2 activity, proving it can be an alternative carrier to an absorbable collagen sponge, with the notable advantages that the hydrogel conforms to the defect better than a collagen sponge and that the hydrogel can be metered more precisely.

The hydrogel was shown to be safe in vitro and in vivo. We were able to confirm that contact between the hydrogel material and tissue did not produce a cytotoxic effect by testing its effect on metabolic activity in a range of representative cell lines. The hydrogel was tested on two types of pre‐osteoblast cell lines, one for humans and one for mice, because the primary site of injection is proximal to bone; neither of which was harmed by the hydrogel. As a way of further confirming its innocuous nature, we tested the hydrogel on MRC‐5 cells, a human lung tissue fibroblast cell line, which showed no adverse effects associated with the hydrogel exposure.

The hydrogel did not a produce a reaction dermally or intracutaneously, as determined using established ASTM standards for testing sensitization of injected materials. In both the rabbit intracutaneous and guinea pig dermal sensitization tests, the hydrogel groups produced very little, if any, erythema or edema, reinforcing the results of the in vitro testing that the hydrogel was non‐inflammatory. Another concern was the degradation of the hydrogel and biodistribution of the degradation products. After conducting an in vivo study where the hydrogel was placed in the calvaria, major organs were excised, sectioned, and examined for evidence of hydrogel particulate. None of the sections showed evidence that, as the hydrogel degraded, its components were not found in any of the major organs, nor did it have a negative impact on their morphology. Based on this study, we conclude that our novel hydrogel has a comparative safety profile to collagen scaffolds and other common drug delivery vehicles.

As a test of biological function in vivo, the hydrogel performed well. Based on our microCT reconstruction and histomorphometric quantifications, the hydrogel produced less total bone growth than the collagen scaffold loaded with rhBMP2. However, the hydrogel was more effective in localizing bone formation induced by BMP2 to the boundaries of the defect. In contrast, collagen + BMP2 caused a significant amount of ectopic bone growth outside the defect (50% of the new bone formed), above the normal parietal bone, which we considered to be heterotopic ossification. The hydrogel was able to limit the amount of heterotopic ossification, which was one of the primary design goals of our system. Based on the histology sections of this bone growth, the BMP2 released from the hydrogel stimulated healthy ossification. Interestingly, hydrogel+BMP2 seemed to do a better job at closing the defect based on the histology. We attribute this increased closure to the fact that it was better able to localize the bone growth. Our results suggest that this may also be due to the formation of new blood vessels throughout the affected site due to the retention of BMP2 within the defect, ultimately leading to new bone.

## 5 Conclusions

Our novel click hydrogel represents a promising alternative to the traditionally used absorbable collagen scaffold typically used to deliver BMP2. Its ability to crosslink rapidly and without a photo initiator or potentially harmful precursor makes it promising for use in a clinical setting. The results presented in this case study demonstrate that the click hydrogel is an effective vehicle for the delivery of BMP2 to orthotopic sites to promote bone regeneration. These findings are supported by our previous studies using the hydrogel to deliver bioactive agents to control the rate of bone growth or to promote osseointegration [20, 21, 22, 23, 24]. The results of the present study indicate that the use of BMP for bone regeneration is enhanced with the use of the gel, with fewer side effects. Further studies will explore the weight‐to‐volume ratio of our crosslinker and the effect on the rate of release of encapsulated biologics as a way to improve the sustained release of BMP2 over the collagen scaffold. Regardless, a clear benefit our hydrogel has over a typical collagen scaffold is its ability to be injected wherever a needle can fit. In terms of bone regeneration treatment, injectability offers immense clinical potential by eliminating invasive surgery to the site of injury to promote healing and warrants further exploration.

## Author Contributions

D.J.C., M.C.M., C.V.D., Z.S., T.W.J., and B.D.B.: Original draft. D.J.C., T.W.J., and Z.S.: Data analysis.

## Disclosure

The patents describing the click hydrogel technology have been licensed by Pascal Medical Corporation (Richmond, VA). B.D.B. is the co‐founder, chief scientific officer, and chairman of the board of Pascal Medical. Z.S., B.D.B., and D.S.W. are co‐inventors of the patents. D.J.C. is a consultant for Pascal. M.C.M. is an employee of Pascal Medical Corporation. This research was supported by grants from the FDA, the NIH, the Commonwealth Research Commercialization Fund, VCU Ventures, and the Virginia Biomedical Health Research Corporation (Catalyst), in addition to gifts from the Joan and Morgan Massey Foundation and Pascal Medical.

## Conflicts of Interest

B.D.B. is Chair, Board of Directors and CEO of Pascal Medical Corporation, which is commercializing the “ClickGel” technology; M.C.M. is an employee of Pascal; D.J.C. is a consultant for Pascal. B.D.B., Z.S., and D.S.W. are co‐inventors of the ClickGel technology. There are no other conflicts.

## Data Availability

Data are available upon written request to B.D.B.
